# Supplementary material for: A flexible and physically transient electrochemical sensor for real-time wireless nitric oxide monitoring
Source: Nat Commun. 2020 Jun 25;11:3207. doi: 10.1038/s41467-020-17008-8 (PMC7316789; doi:10.1038/s41467-020-17008-8)
Supplement: Supplementary file 1 — Supplementary Information [file 41467_2020_17008_MOESM1_ESM.pdf]

Supplementary Information

A flexible and physically transient electrochemical sensor for real-time wireless nitric oxide monitoring

by Li et al.

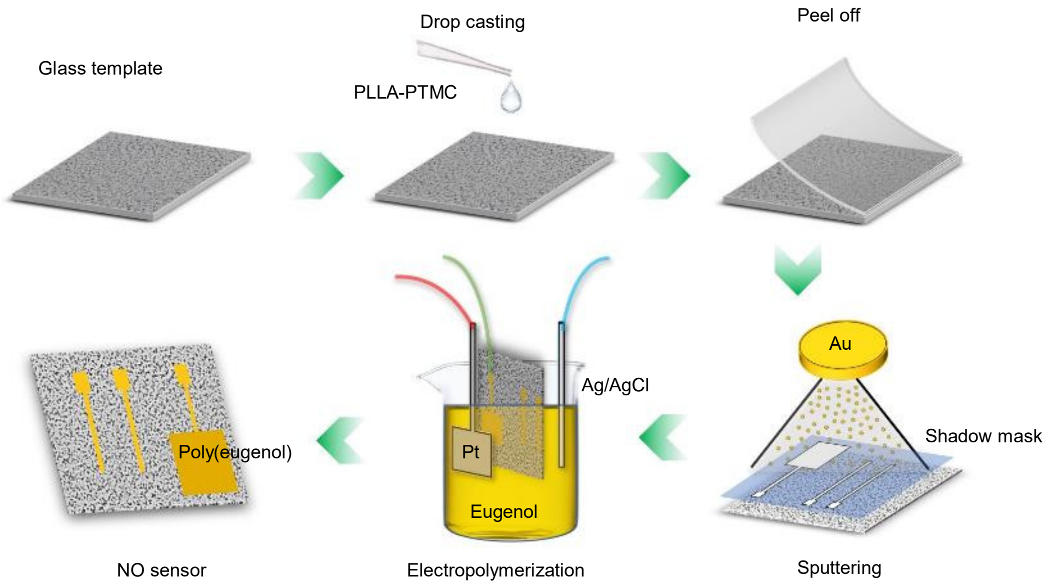

Supplementary Figure 1: Fabrication process of NO sensors

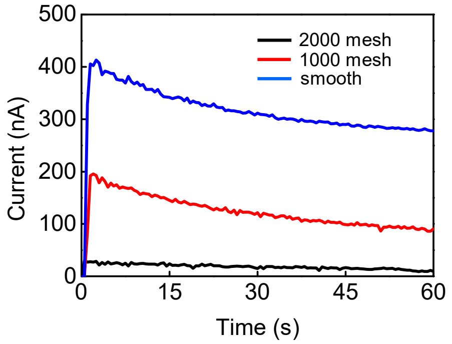

Supplementary Figure 2: Current response of Au electrodes fabricated with different substrate templates in phosphate buffered saline (PBS) at 37 °C. Black line: smooth glass template; red line: 1000 mesh glass template; blue line: 2000 mesh glass template. *n* = 3 independent experiments.

(a) (b) (c)

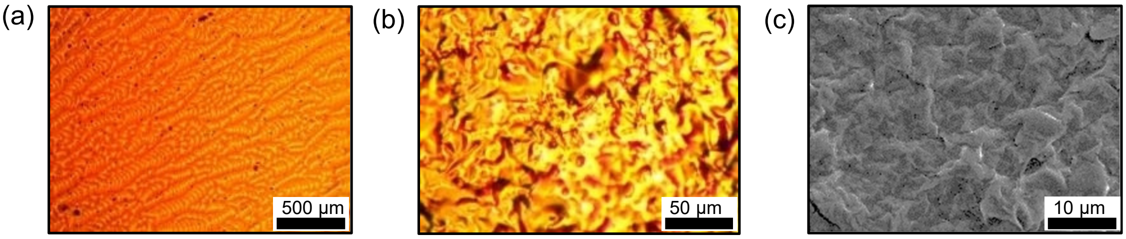

Supplementary Figure 3: Surface morphology of Au electrodes. (a) Optical image (50X). (b) Optical image (600X). (c) SEM image (1000X).

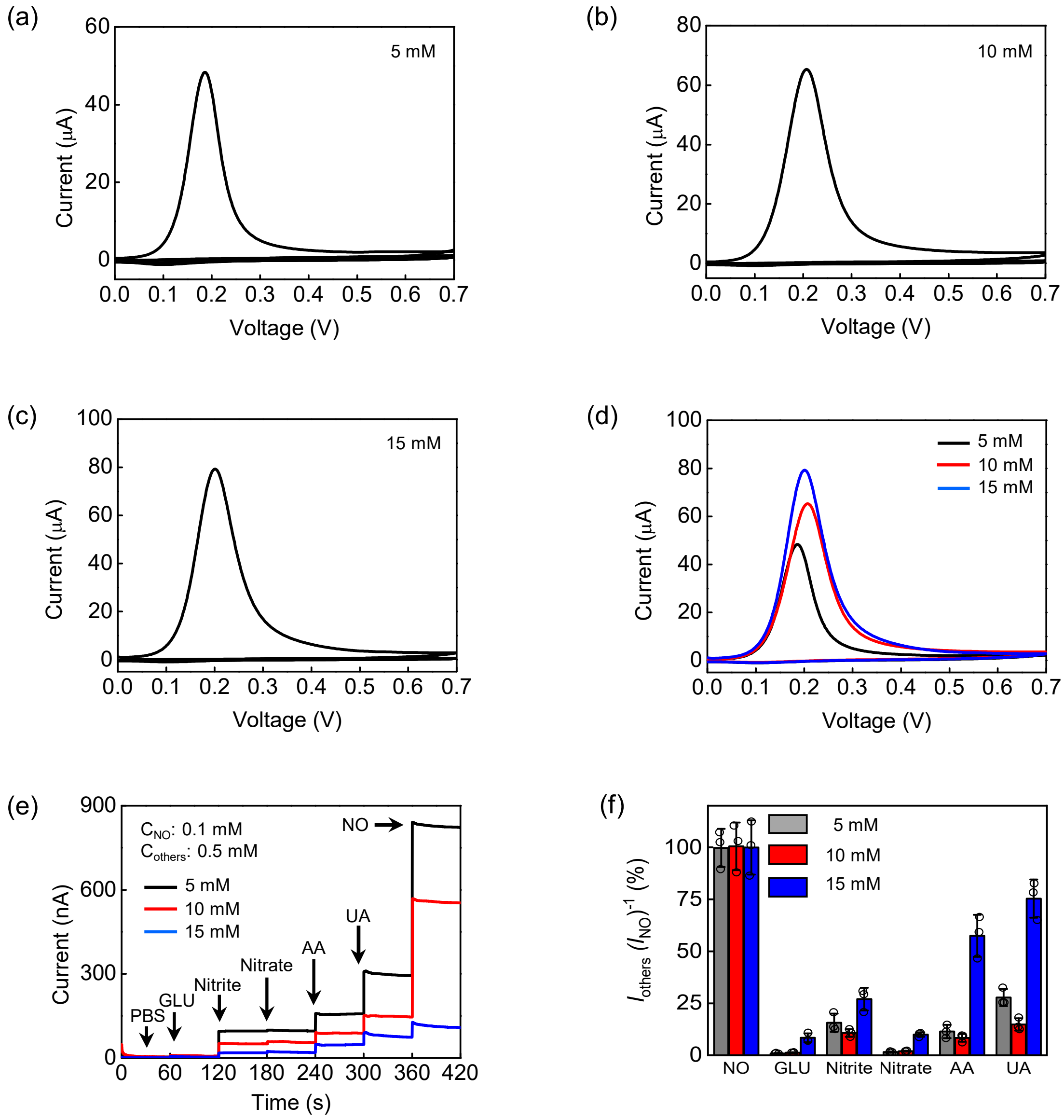

Supplementary Figure 4: Preparation and characterization of selective poly(eugenol) membrane of the NO sensor. (a) Electropolymerization curves with 5 mM eugenol solution. (b) Electropolymerization curves with 10 mM eugenol solution. (c) Electropolymerization curves with 15 mM eugenol solution. (d) The first cyclic voltammetry cycle of eugenol electropolymerization (5, 10, 15 mM). (e) Selectivity of NO sensors performed at 37 °C with different thickness of poly(eugenol) layers (electropolymerization with 5, 10, 15mM eugenol solutions). (f) Quantitative analysis of the selectivity with different thickness of poly(eugenol) layers (electropolymerization with 5, 10, 15mM eugenol solutions). In a–f, n =3 independent experiments. In f, data are shown as means ± standard deviations.

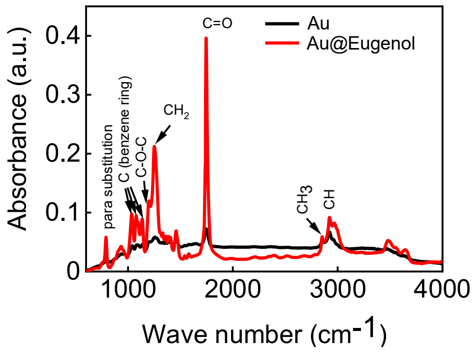

Supplementary Figure 5: FTIR curves of the Au electrodes of NO sensors before and after eugenol electropolymerization. *n* =3 independent experiments.

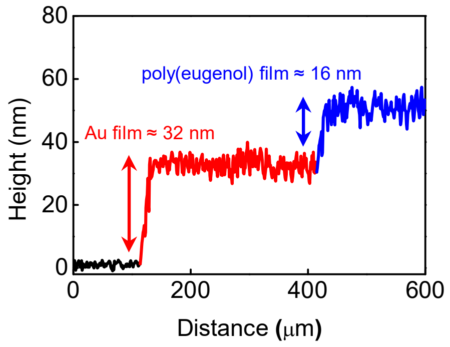

Supplementary Figure 6: Height profile of the working electrode of a NO sensor. $n = 3$ independent experiments.

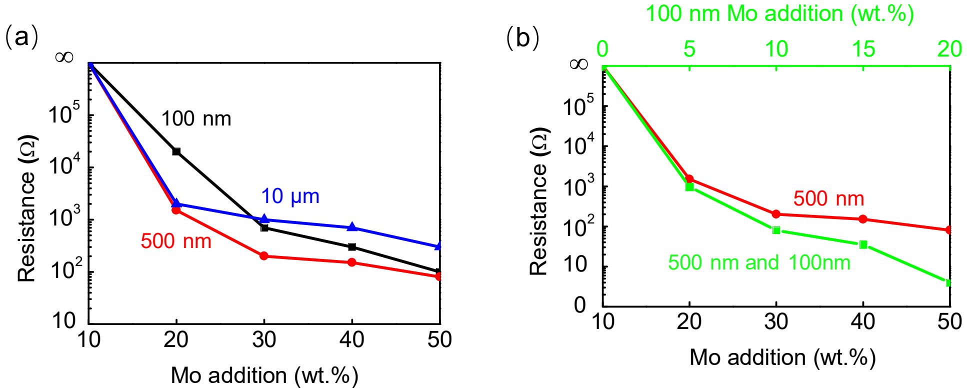

Supplementary Figure 7: Resistance of biodegradable paste made of Mo particles and PLLA-PTMC. (a) Resistance of the biodegradable paste with different Mo particle sizes and concentrations. (b) Resistance of biodegradable paste mixing different concentrations of 100 nm Mo and 500 nm Mo. $n = 3$ independent experiments.

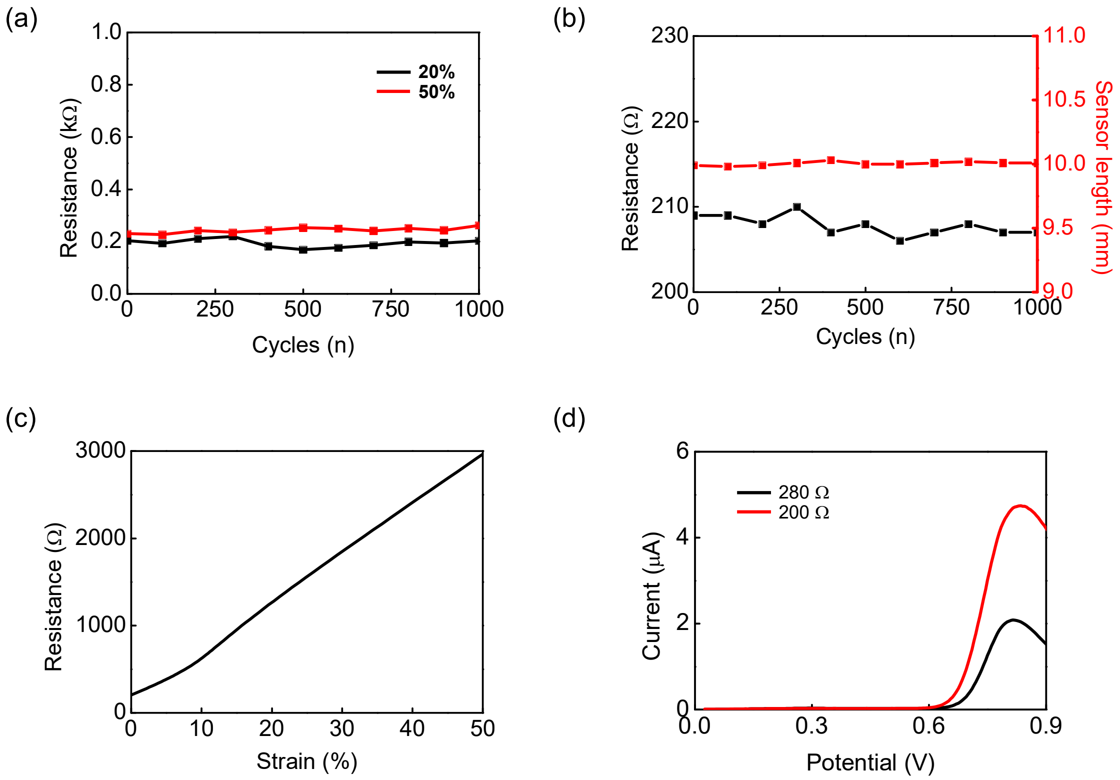

Supplementary Figure 8: (a) Resistance of Au electrodes of tensile tests with the strain of 20% and 50% for 1000 cycles. (b) Resistance and length of Au electrodes of bend tests at angels up to 90 degree for 1000 cycles. (c) Resistance of Au electrodes as a function of strain upon stretching. (d) Response current to NO with Au electrodes of different resistance. In a and b, $n = 3$ independent experiments.

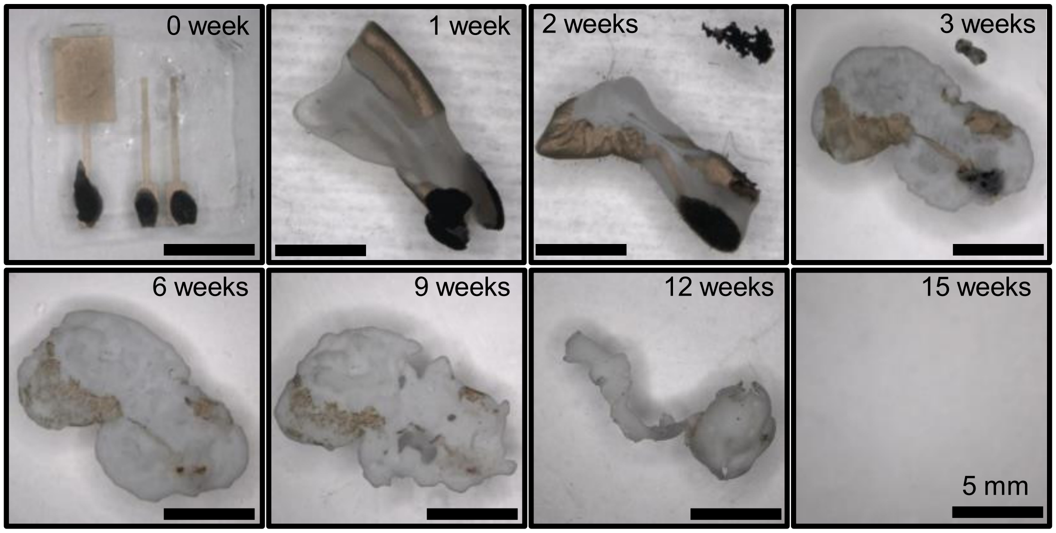

Supplementary Figure 9: Degradation process of a NO sensor at various stages in PBS at 65 ^{o}C for 15 weeks.

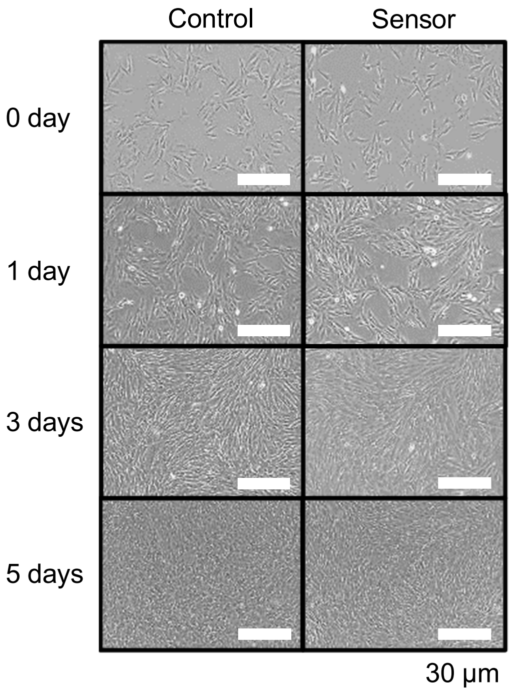

Supplementary Figure 10: Optical images of human aortic vascular smooth muscle cells (HA-VSMCs) proliferation of the sensor group (cells co-incubated on the NO sensor) and the control group. $n = 3$ independent experiments.

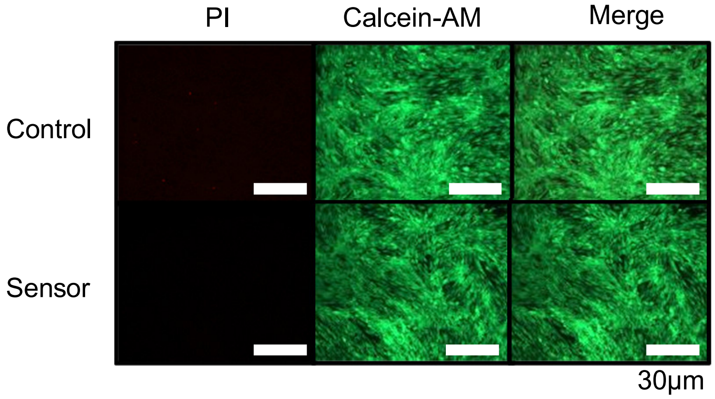

Supplementary Figure 11: Florescent images of HA-VSMCs proliferation of the sensor group (cells co-incubated on the NO sensor) and the control group on the 5th day. Propidium Iodide (PI) staining (red) for dead cells and Calcein-AM staining (green) for living cells. *n* = 3 independent experiments.

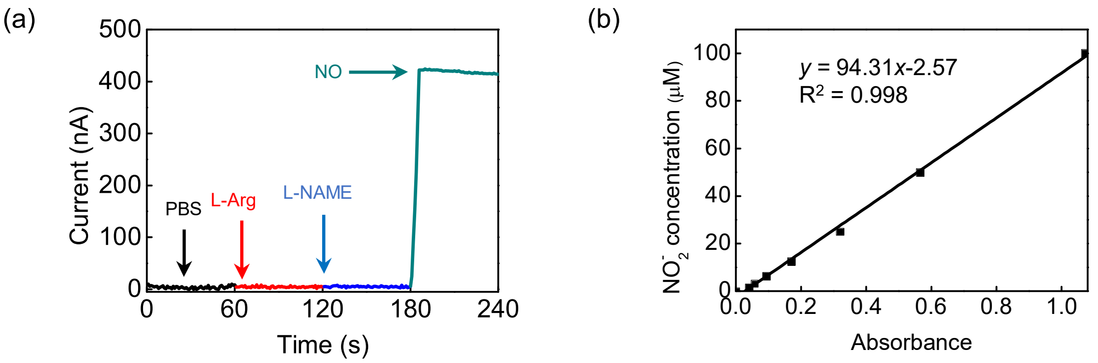

Supplementary Figure 12: (a) Selectivity measurement in PBS: current response with the additions of L-arginine (L-Arg) (5 mM) and N^{ω}-nitro-L-arginine methyl ester (L-NAME) (10 mM) and NO solutions (0.1 mM). (b) Calibration curve of Griess test.

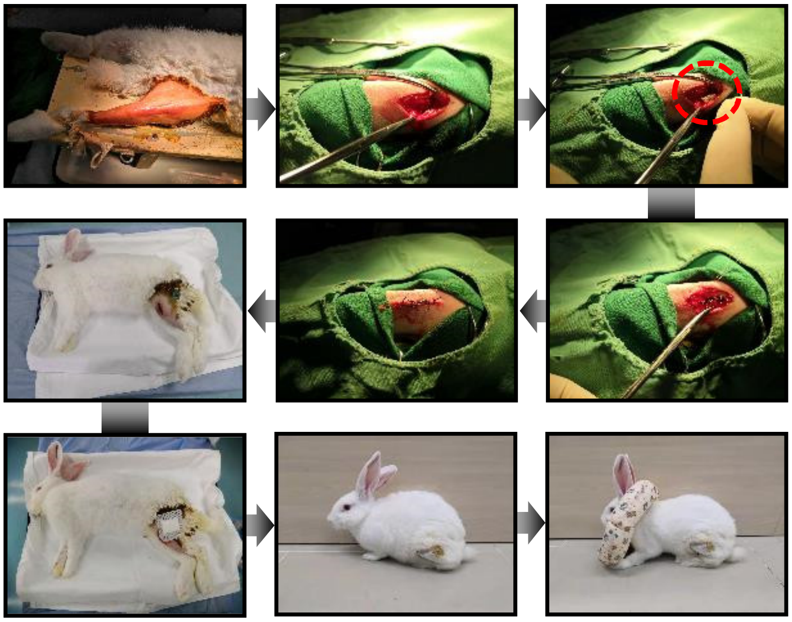

Supplementary Figure 13: Surgical operation process of NO sensor implantation.

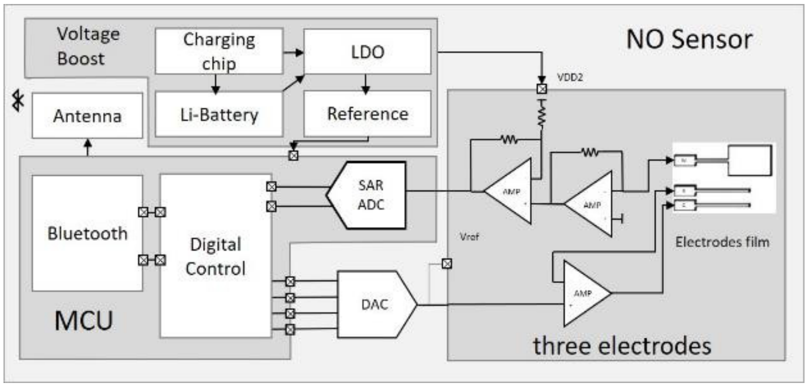

Supplementary Figure 14: Schematic diagram of wireless control and transmission system for the NO sensor.

(a) (b)

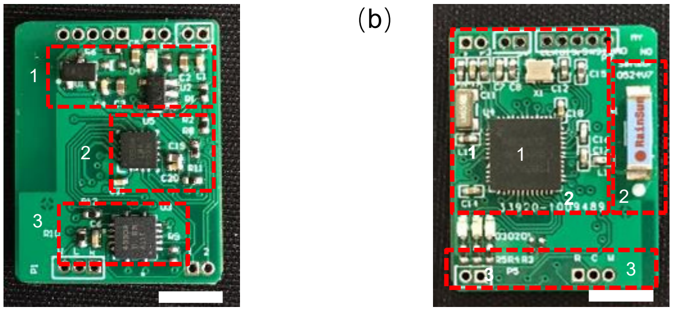

5 mm

Supplementary Figure 15: Images of system architecture for NO sensor. (a) 1-Battery management system (BMS); 2-Digital to Analog Converter (DAC); 3-Amplifier (AMP). (b) 1-Analog to Digital Converter (ADC), General Purpose Input/Output (GPIO), Serial Peripheral Interface (SPI); 2-Antenna; 3-Test pin.

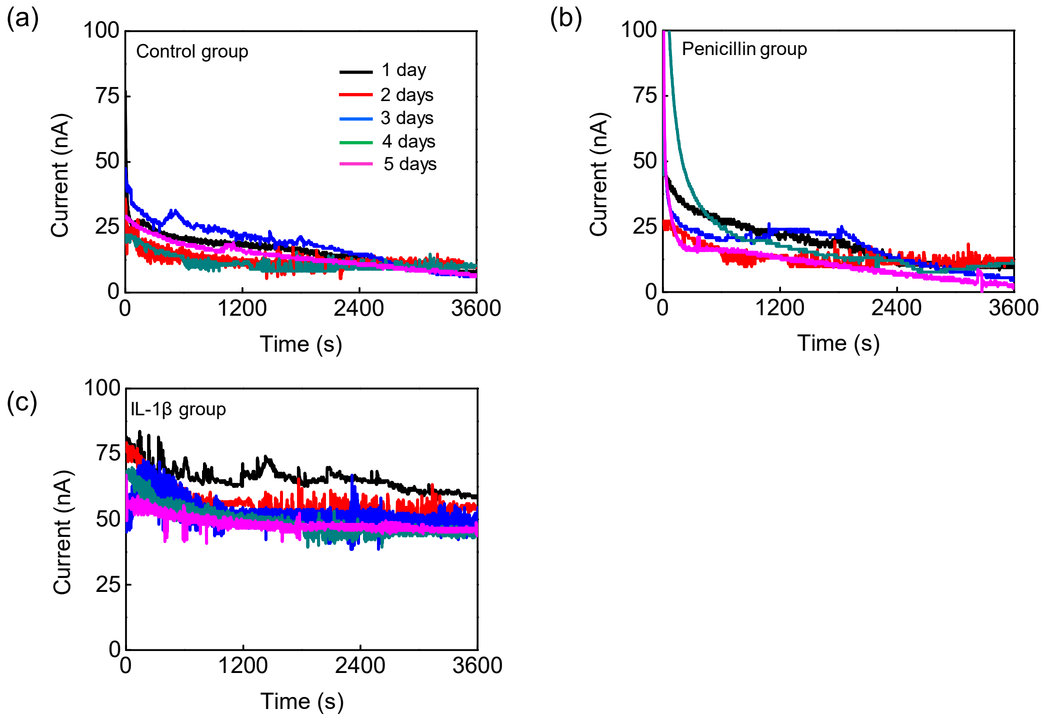

Supplementary Figure 16: Real-time monitoring of the current response to NO in the joint cavity of a New Zealand Rabbit. Data are recorded for one hour everyday for a 5-day period. (a) Control group (sensor implantation without treatment). (b) Penicillin group (antibiotic treatment after sensor implantation). (c) Interleukin-1 beta (IL-1β) group (promoting inflammation after sensor implantation). In a-c, *n* = 3 independent experiments.

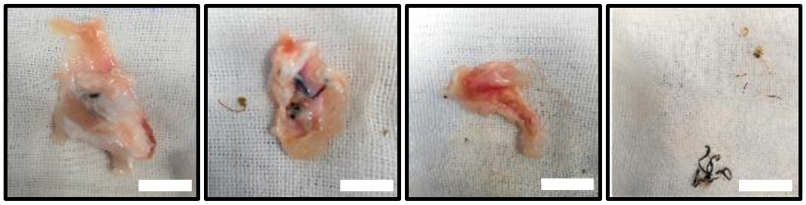

Supplementary Figure 17: Anatomical images of the surrounding tissue at the implantation site of the NO sensor after 8 weeks. *n* = 3 independent experiments.

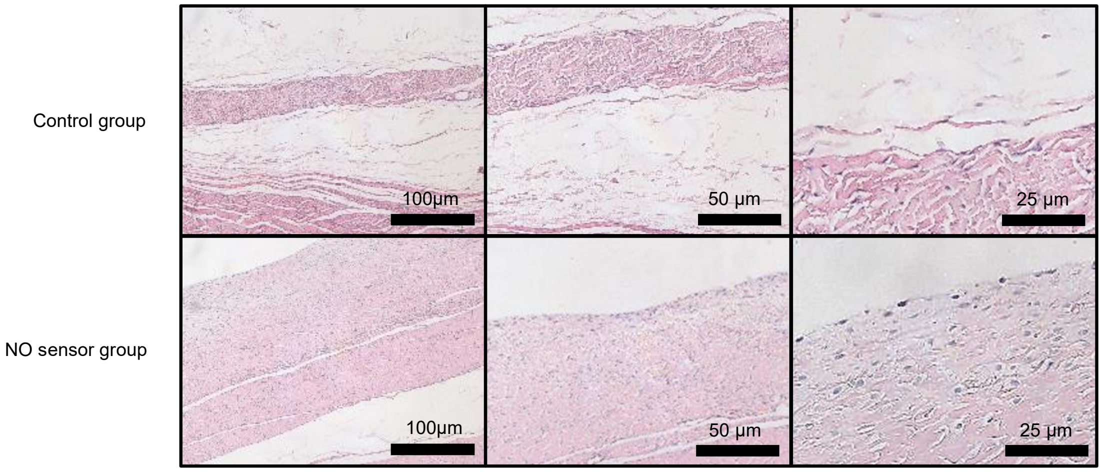

Supplementary Figure 18: Hematoxylin-eosin (HE) staining images of tissues surrounding the implantation site of the NO sensor. Sensor group: with NO sensor implantation. Control group: without implantation. *n* = 3 independent experiments.

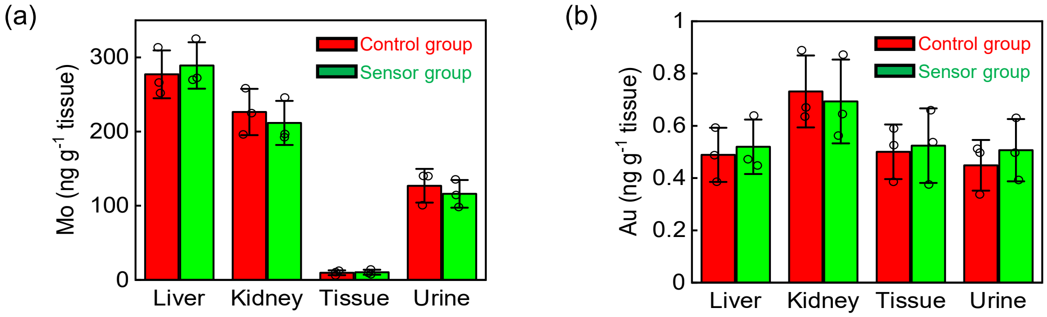

Supplementary Figure 19: ICP-MS results showing Mo and Au concentrations of the tissues at the implantation site and various organs of the New Zealand Rabbit after 8 weeks of implantation. Sensor group (green): with NO sensor implantation. Control group (red): without implantation. (a) Mo concentration; (b) Au concentration. $n = 3$ independent experiments. Data are shown as means ± standard deviations.

Supplementary Table 1: Comparison of the performance of amperometric NO sensors from the previously reported work and the current work.

Sensor type	Working potential (V)	Sensitivity (nA/μM)	Linear dynamic range (μM)	Detection limit (nM)	Response time (s)	Applications	Flexibility and Degradability	In vivo test	Ref
Carbon fiber/ Nafion/o-PD	0.74 vs. SCE	9.60	0.02-0.2	36	-	NO solution	No	No	1
Au fiber/ Nafion	0.68 vs. SCE	0.8	10-100	-	-	NO solution	No	No	2
AuNPs/GO	0.8 vs. SCE	27.5	0.036-70	18	-	Fish liver homogenate	No	No	3
AuNPs/ 3D GH	0.81 vs. Ag/AgCl	45.27	0.2-6	9	2.92	JB6-C30 cells B16-F10 cells	No	No	4
PEBT	0.8 vs. Ag/AgCl	356.4	0.1-99	36	3	Rat heart homogenate	No	No	5
Pt/ fluorinated xerogel	0.85 vs. Ag/AgCl	0.106 ± 0.028	0-3.13	9.55	3.7 ±0.7	Rat cortical deep-layer	No	Yes	6
Hemin/Carbon nanotubes/Chitosa n	-0.76 vs. Ag/AgCl	1.72	0.25-1	25	-	Rat brain	No	Yes	7
FGPC/AuNPs	0.78 vs. Ag/AgCl	357	0.005-0.2	3.2		HUVECs Rat acupoints	No	No	8
WPI ISO-NOP		2	-	1	< 5	Cell culture	No	No	9
Rough Au film/ Poly(eugenol)	~ 0.8 vs. Au	5.29 (0.01-5μM) 4.17 (5-100μM)	0.01-100	3.97	< 0.35	Rat chondrocyte; Rat and rabbit kidney, liver, heart and brain; Rat heart and joint cavity	Yes	Yes	This work

Supplementary References:

1 Pontie, M., Bedioui, F. & Devynck, J. New composite modified carbon microfibers for sensitive and selective determination of physiologically relevant concentrations of nitric oxide in solution. *Electroanal* **11**, 845-850, (1999).

2 Bedioui, F., Trevin, S. & Devynck, J. The Use of Gold Electrodes in the Electrochemical Detection of Nitric-Oxide in Aqueous-Solution. *J Electroanal Chem* **377**, 295-298, (1994).

3 Wang, Y. Z., Song, B., Xu, J. H. & Hu, S. S. An amperometric sensor for nitric oxide based on a glassy carbon electrode modified with graphene, Nafion, and electrodeposited gold nanoparticles. *Microchim Acta* **182**, 711-718, (2015).

4 Li, J. L., Xie, J. L., Gao, L. X. & Li, C. M. Au Nanoparticles-3D Graphene Hydrogel Nanocomposite To Boost Synergistically in Situ Detection Sensitivity toward Cell-Released Nitric Oxide. *Acs Appl Mater Inter* **7**, 2726-2734, (2015).

5 Geng, M. J., Xu, J. H. & Hu, S. S. In situ electrogenerated poly(Eriochrome black T) film and its application in nitric oxide sensor. *React Funct Polym* **68**, 1253-1259, (2008).

6 Moon, J. *et al.* Dual Electrochemical Microsensor for Real-Time Simultaneous Monitoring of Nitric Oxide and Potassium Ion Changes in a Rat Brain during Spontaneous Neocortical Epileptic Seizure. *Anal Chem* **88**, 8942-8948, (2016).

7 Santos, R. M., Rodrigues, M. S., Laranjinha, J. & Barbosa, R. M. Biomimetic sensor based on hemin/carbon nanotubes/chitosan modified microelectrode for nitric oxide measurement in the brain. *Biosens Bioelectron* **44**, 152-159, (2013).

8 Tang, L. *et al.* A sensitive acupuncture needle microsensor for real-time monitoring of nitric oxide in acupoints of rats. *Sci Rep* **7**, (2017).

9 https://www.wpiinc.com/biosensing/biosensors/nitric-oxide-sensors.
