# Supplementary material for: A flexible and physically transient electrochemical sensor for real-time wireless nitric oxide monitoring
Source: Nat Commun. 2020 Jun 25;11:3207. doi: 10.1038/s41467-020-17008-8 (PMC7316789; doi:10.1038/s41467-020-17008-8)
Supplement: Supplementary file 3 — Description of Additional Supplementary Files [file 41467_2020_17008_MOESM3_ESM.docx]

Description of Additional Supplementary Files

**Title:** Supplementary Movie 1.

**Description:** Simultaneous electrocardiography (ECG) signal recording upon NO detection using NO sensors in the heart region of a rabbit. The bottom signals indicate arrhythmia.

**Title:** Supplementary Movie 2.

**Description:** Wireless data transmission of the response current with the addition of NO solutions to a mobile device.
